# Histological, Biomechanical, and Biological Properties of Genipin-Crosslinked Decellularized Peripheral Nerves

**DOI:** 10.3390/ijms22020674

**Published:** 2021-01-12

**Authors:** Óscar Darío García-García, Marwa El Soury, David González-Quevedo, David Sánchez-Porras, Jesús Chato-Astrain, Fernando Campos, Víctor Carriel

**Affiliations:** 1 Tissue Engineering Group, Department of Histology, University of Granada, 18016 Granada, Spain; e.oscargg@go.ugr.es (Ó.D.G.-G.); ymarwa.elsoury@unito.it (M.E.S.); davidgonzalesq.sspa@juntadeandalucia.es (D.G.-Q.); davidsp@go.ugr.es (D.S.-P.); jchato@go.ugr.es (J.C.-A.); 2 Instituto de Investigación Biosanitaria ibs. GRANADA, 18012 Granada, Spain; 3 Doctoral Program in Biomedicine, University of Granada, 18012 Granada, Spain; 4 Department of Clinical and Biological Sciences and Neuroscience Institute Cavalieri Ottolenghi (NICO), University of Torino, 10043 Orbassano, Italy; 5 Department of Orthopedic Surgery and Traumatology, Regional University Hospital of Málaga, 29010 Málaga, Spain

**Keywords:** tissue engineering, nerve repair, nerve tissue decellularization, genipin, chemical crosslinking, histology, natural biomaterials, biomechanical and structural properties, cell-biomaterials interactions

## Abstract

Acellular nerve allografts (ANGs) represent a promising alternative in nerve repair. Our aim is to improve the structural and biomechanical properties of biocompatible Sondell (SD) and Roosens (RS) based ANGs using genipin (GP) as a crosslinker agent ex vivo. The impact of two concentrations of GP (0.10% and 0.25%) on Wistar rat sciatic nerve-derived ANGs was assessed at the histological, biomechanical, and biocompatibility levels. Histology confirmed the differences between SD and RS procedures, but not remarkable changes were induced by GP, which helped to preserve the nerve histological pattern. Tensile test revealed that GP enhanced the biomechanical properties of SD and RS ANGs, being the crosslinked RS ANGs more comparable to the native nerves used as control. The evaluation of the ANGs biocompatibility conducted with adipose-derived mesenchymal stem cells cultured within the ANGs confirmed a high degree of biocompatibility in all ANGs, especially in RS and RS-GP 0.10% ANGs. Finally, this study demonstrates that the use of GP could be an efficient alternative to improve the biomechanical properties of ANGs with a slight impact on the biocompatibility and histological pattern. For these reasons, we hypothesize that our novel crosslinked ANGs could be a suitable alternative for future in vivo preclinical studies.

## 1. Introduction

The peripheral nerves (PNs) are delicate conductive organs that ensure the motor, sensory, and autonomic communication between the central nervous system and distal target organs [1]. These organs have two components, the nerve tissue and the connective tissue or stroma. The nerve tissue is represented by conductive functional units called PN fibers which can be myelinated or unmyelinated [1]. In the case of the stroma, it is composed by three layers, the epineurium, the perineurium, and the endoneurium, and they confer the structural and biomechanical properties to these crucial organs [1,2].

The large extension of the PNs make them vulnerable to a wide range of PN traumatic injuries at any anatomic site [1]. The most common PN traumatic injuries are nerves compressions and/or tractions, lacerations, incomplete or complete nerve transections, or avulsion of spinal nerves roots [3,4]. The estimated incidence of the PN traumatic injuries is upward 300,000 cases per year in Europe [5] and more than one million per year worldwide [6]. In addition, the surgical dissection of primary or metastatic cancers can also severely affect the structure and function of the PNs creating complex defects that need to be repaired [7].

Direct peripheral nerve repair is the preferred treatment in short nerve defects, and autologous nerve grafting is the current gold standard technique for the reconstruction of severe injuries [1]. However, nerve autograft technique is effective in approximately 50% of the cases and there are several well-known disadvantages limiting their use. In this context, nerve allografts started to be used with variable success. They provide an adequate extracellular matrix (ECM) and cells, but also maintain an active host immune response with a high risk of graft rejection [8,9] and, therefore new alternatives for these patients are still needed [10,11,12,13,14]. In order to respond to these needs, different kind of engineered substitutes and/or biomedical devices for peripheral nerve repair have been generated by tissue engineering. Among these products, the generation and use of acellular nerve grafts (ANGs) has proved to be one of the most promising strategies in the field, those being generated with the method described by Sondell, one of the most widely used in the field.

Tissue decellularization technique was designed to efficiently remove all cellular material, to reduce the immunogenicity, from a specific tissue or organ while preserving the molecular composition, biological activity, 3D structure, and biomechanical integrity of the remaining ECM [15,16]. These ANGs provide an adequate and tissue-specific ECM to the regenerative microenvironment explaining its promising results and pro-regenerative properties [17]. However, an ideal decellularization technique does not yet exist and therefore a certain and irreversible degree of chemical, structural, or even biological compromise of the remaining ECM always occurs [18,19]. Adequate decellularization and ECM preservation was achieved in nerve tissue engineering with the protocol developed by Roosens (RS) [20] and subsequently adapted for nerve [21], which showed superior ex vivo properties than the ANGs generated by Sondell (SD) or Hudson-based (HD) technique [21]. As expected, the impairment of the ECM, due to the decellularization process, resulted in a negative impact on overall biomechanical properties and structural stability of the ANGs generated [16,18]. In effect, the in vivo evaluation of these products revealed that the ANGs (RS, SD, and HD) were more delicate and difficult to handle during surgery than the nerve autograft techniques [22]. Despite these findings, this in vivo study revealed a degree of regeneration and functional recovery comparable to, but not superior to, the use of autografting, especially when the new RS-ANGs were used [22].

An efficient manner to improve the biomechanical properties and structural stability of acellular ECM is the use of chemical crosslinking procedures [23]. These agents can induce, under certain circumstances, inter or intramolecular covalent interactions between the ECM molecules or biomaterials [24]. In tissue engineering, several chemical agents have been used being the glutaraldehyde one of the most widely used for this purpose. Nevertheless, despite its acceptable efficiency, a number of disadvantages limit its use (such as cytotoxicity, low in vivo biocompatibility, dystrophic calcification, suboptimal cell attachment, and growth) [25,26,27]. In recent years, genipin (GP), a natural iridoid glycoside, extracted and purified from the *Gardenia Jasminoides* has emerged as an alternative to glutaraldehyde [28]. This agent was successfully used for the crosslinking of engineered biomaterials (chitosan, collagen, fibrin-agarose, etc.) and decellularized ECM (pericardial tissue, heart valves, cornea, spinal cord, blood vessels, etc.) [29,30,31,32,33,34,35] with an evident improvement of the biomechanical properties and better biocompatibility and biological properties than glutaraldehyde [28,32,35]. Nevertheless, higher concentrations of GP are not recommended because they may decrease the biological properties and increase the rigidity and fragility of the biomaterials [32]. In addition, other studies suggest that GP has notable anti-inflammatory properties that make it an attractive alternative for use with ANGs [36,37,38].

In this sense, several studies in nerve tissue engineering support that ANGs are one of the most promising alternatives for peripheral nerve repair. In fact, these grafts provide a unique natural and tissue-specific ECM, difficult to generate with the current tissue engineering biofabrication processes, which resolve some of the limitations associated to auto or allograft techniques. Nevertheless, their structural and biomechanical weakness, an irreversible consequence of the decellularization process, remain a challenge. However, they could be potentially restored or even improved with the use of GP. This agent is capable of spontaneously reacting with primary amino groups (mainly of lysine, hidroxylisine, or arginine residues) inducing intra and intermolecular crosslinking in the collagen network, which are the main ECM molecules that remain after nerve tissue decellularization [21,22,39]. For this reason, the aim of this study was to explore the possibility of chemically improving the biomechanical properties of ANGs for a more efficient and safe use in peripheral nerve repair. In this context, the impact of two efficient and biologically safe concentrations of GP (0.10% and 0.25%) on the histological, ultrastructural, biomechanical, and biological properties of the promising RS [21] and the classical SD [40] ANGs, were investigated ex vivo.

## 2. Results

### 2.1. Histology and Quantitative Histochemistry

**Figure 1:**
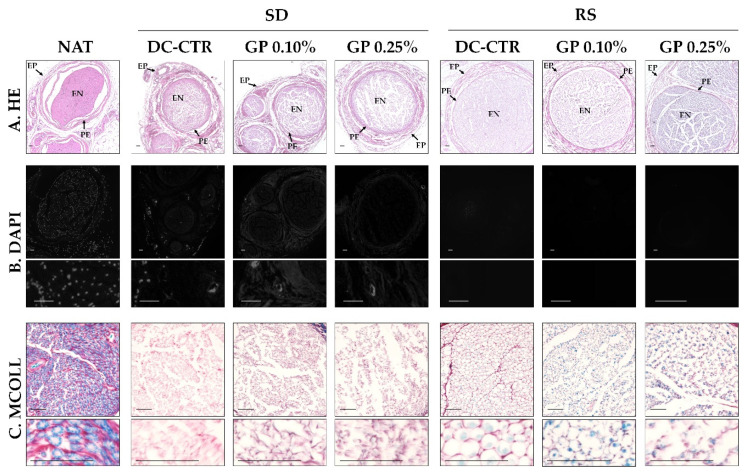
Light and fluorescent microscopy of native (NAT) and acellular nerve allografts (ANGs) generated by Sondell (SD) and Roosens (RS) protocols. DC-CTR indicate the SD or RS non-crosslinked ANGs, while GP 0.10 and GP 0.25% correspond to the concentrations of genipin (GP) used within ANGs (**A**) Sections stained with hematoxylin eosin or HE. In HE images epineural (EP), perineurial (PE), and endoneurial (EN) compartments are indicated. (**B**) Fluorescent microscopy images of nerves stained with the intercalant fluorochrome 4´,6-diamidino-2-phenylindole (DAPI) that recognizes the A-T interactions of DNA. (**C**) Histological sections stained with the myelin-collagen histochemical method (MCOLL) for the identification of myelin (light-blue) and collagen fibers (red). Scale bar = 100 µm.

Regarding the histological characterization of the ANGs generated, hematoxylin & eosin (HE) analyses revealed that the general PNs histological structure was relatively well-preserved after decellularization and subsequent crosslinking with GP (Figure 1). Native nerves (used as control) showed their characteristic stromal organization with well-organized epineural, perineurial, and endoneurial compartments. After SD and RS decellularization processes, some structural changes were observed. In the case of SD groups, HE staining showed a clear disruption of the endoneurial compartment, especially with the use of GP, but perifascicular connective tissue layers were less affected (Figure 1). The analysis of RS groups revealed a better preservation of endoneurial compartment with a well-defined perineurial layer and surrounding epineurial connective tissue. No differences were observed with the use of GP except the blue background color due to the action of 0.25% GP. In relation to the nuclei preservation, HE and especially DAPI staining did not reveal the presence of well-preserved nuclei, but few traces of DNA were found especially in SD group (Figure 1).

Myelin and surrounding collagen network were evaluated by using a histochemical method for myelin and collagen simultaneous identification (MCOLL). This technique confirmed the characteristic and abundant myelination and well-delignated collagen content in healthy nerves (Figure 1). No positive histochemical reaction for myelin was observed in SD groups but this method revealed a thin endoneurial collagen network. Interestingly, certain positivity for myelin was perceived in RS groups. The myelin was observed immersed in a well-preserved collagen endoneurial ECM in RS-CTR. Myelin reaction was slightly more evident in RS-GP groups where the collagen ECM was less organized (Figure 1).

**Figure 2:**
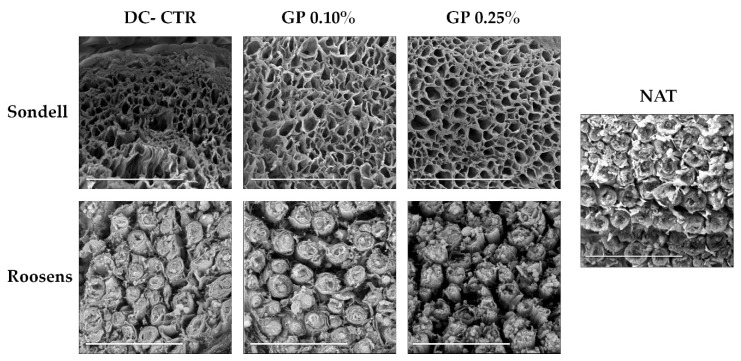
Scanning electron microscopy of native and ANGs. Note the 3D organization of the remaining extracellular matrix (ECM) in ANGs which in the case of Sondell groups was possible to identify the endoneurial tubes, especially after the chemical crosslinking with GP. In the case of Roosens groups, this technique confirmed the presence of myelin remnants at the endoneurial compartment). Scale bar = 10 µm.

Scanning electron microscopy (SEM) ultrastructural analyses showed the dense and highly compact structure of the endoneurial compartment in native nerves. This technique showed that SD method was able to remove the PN fibers leaving an irregular and poorly structured endoneurial tubes (Figure 2). In addition, the use of GP, especially at 0.25%, improved the structure and homogeneity of the remaining endoneurial tubes (Figure 2). In the case of the ANGs generated with RS method, SEM confirmed the preservation of the endoneurial tubes filled by irregular remnants of myelin. The definition of these structures was improved with the use of GP without differences between the GP concentrations used. Moreover, SEM evaluation did not allow us to determine if axons, cells, or other cellular component persisted after decellularization and subsequent GP crosslinking (Figure 2).

**Figure 3:**
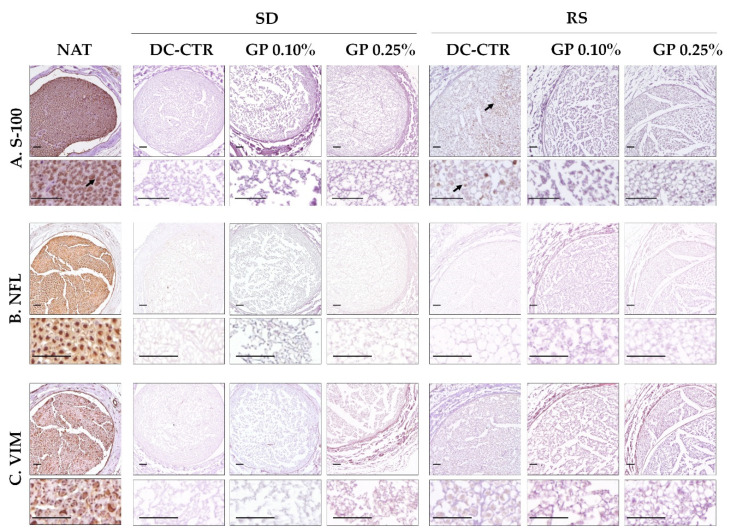
Immunohistochemical evaluation of cellular and cytoskeletal proteins remnants in ANGs. These analyses confirmed the efficient removal of the cellular components evaluated: (**A**). S-100, (**B**). neurofilament (NFL) and (**C**). vimentin (VIM) followed PNs decellularization. For each experimental condition and technique high magnification images were included. Note thata slight S-100 positive reaction persisted in RS group (indicated with black arrows). The typical immunohistochemical pattern for each marker could be observed in native nerves used as control (NAT). Scale bar = 200 µm.

In order to determine if some cellular protein remnants persisted after decellularization immunohistochemistry for S-100, neurofilament (NFL) and vimentin (VIM) were conducted (Figure 3). The positive reaction for these proteins was found with the typical distribution in native nerves. When these proteins were evaluated in ANGs and crosslinked ANGs, no immunohistochemical reactions for Schwann cells (S-100 and vimentin), axons (neurofilament), and fibroblast (vimentin) were observed, except in RS-CTR group where a weak and irregularly distributed reaction for S-100 was observed (Figure 3).

**Figure 4:**
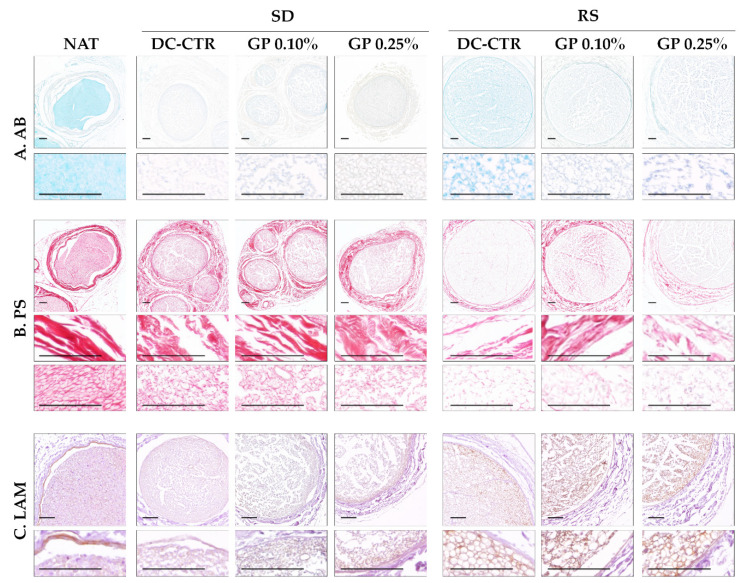
Histochemical and immunohistochemical analysis of the remaining ECM molecules in ANGs. (**A**) Shows the positive histochemical reaction of acid proteoglycans (light-blue) by Alcian blue (AB) histochemical method. (**B**) Histochemical identification of fibrillar collagens (red) by using Picrosirius (PS) staining. (**C**) Immunohistochemical identification of the basal membrane glycoprotein laminin (LAM) at the endoneurial tubes level (brown positive reaction), which were well-preserved in the RS groups. High magnification images were included for each condition and staining. Scale bar = 100 µm.

**Figure 5:**
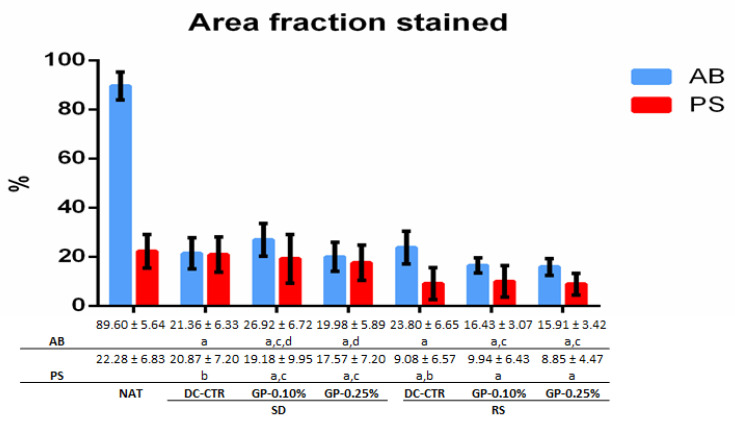
Quantitative histochemical analyses of alcian blue (AB) and Picrosirius (PS) stainings in native nerve and ANGs. Graphical and numeric representation of the area fraction mean values ± standard deviation values (error bars) corresponding to each histochemical positive reaction/histological area in each experimental condition and native nerves used as control (NAT). Statistically significant differences (*p* < 0.05) were determined with the Mann–Whitney test and represented as follows: a = statistically significant differences vs. NAT group. b = statistically significant differences between SD-CTR vs. RS-CTR groups. c = statistically significant differences vs. its corresponding DC-CTR group. d = statistically significant differences between GP 0.10% vs. GP 0.25% concentration used in each decellularized condition (SD or RS).

To evaluate the preservation of ECM components, histochemical and immunohistochemical assays were carried out. Alcian blue (AB) staining (Figure 4) showed the presence of acid proteoglycans at the intrafascicular level in native nerves. These non-fibrillar ECM molecules were efficiently removed from all histological layers with SD method without histochemical differences after GP crosslinking. In relation to RS groups, AB staining showed a partial preservation of these molecules, being especially well-defined in the perineurium (Figure 4). Curiously, in RS-GP groups a decrease in the distribution of the AB histochemical reaction was observed (Figure 4). These findings were corroborated by the quantitative analysis of the area occupied by AB histochemical reaction at the endoneurial level (Figure 5). In this context, all ANGs showed significantly lower area fraction values for AB as compared to the native condition (*p* < 0.05). In addition, no statistical differences were found between SD-CTR and RS-CTR groups (*p* = 0.13). Interestingly, SD-GP 0.10% AB area fraction values were significantly higher (*p* < 0.05) than SD-CTR and SD-GP 0.25% groups (Figure 5). In contrast, the use of both GP concentrations within RS ANGs resulted in a significant decrease of the AB area fraction values as compared with RS-CTR group (*p* < 0.05, Figure 5).

When the collagen network was analyzed by Picrosirius (PS) staining, a relatively well preservation of these fibers was observed after both decellularization procedures (Figure 4). In SD groups, the epi, and perineurial collagen ECM lost structural organization and compaction, while it was moderately well preserved at the endoneurial level. The evaluation of RS-CTR and RS-GP groups showed a decrease of the PS histochemical reaction in all histological compartments (Figure 4). Despite this decrease, PS revealed the preservation of well-defined collagen endoneurial tubes in RS-CTR and especially RS-GP groups (Figure 4). These results were clarified by the PS area fraction values (Figure 5). Surprisingly, no statistical differences were observed between native nerves and SD-CTR group. Moreover, SD-GP, RS-CTR, and RS-GP groups presented significantly lower PS area fraction values than native condition (*p* < 0.05, Figure 5). The decrease of PS area fraction values obtained with the use of both GP concentrations as compared to SD-CTR were significant (*p* < 0.05), but no statistical significant differences were obtained between RS-CTR and RS-GP groups (*p* > 0.05, Figure 5).

Finally, the immunohistochemical analysis of laminin (LAM) confirmed the preservation of this basal lamina glycoprotein delineating the endoneurial tubes in RS groups, without any impact of GP treatment on these immunohistochemical reactions. Curiously, we did not observe any immunohistochemical reaction for laminin in SD-CTR and SD-GP groups (Figure 4).

### 2.2. Quantification of DNA Content

**Figure 6:**
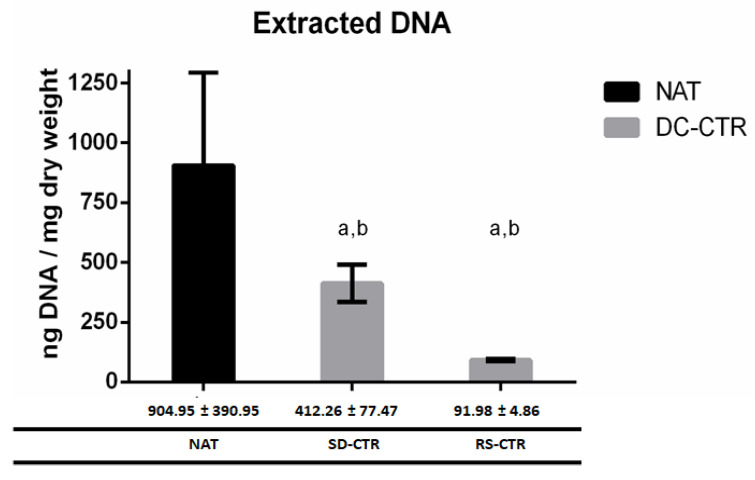
Graphic and numeric representation of the content of DNA in native and ANGs. Extracted DNA is represented as mean ± standard deviation values and statistically significant differences (*p* < 0.05) were determined with Mann–Whitney test as follow: a = statistically significant differences vs. NAT group. b = statistically significant differences between SD-CTR vs. RS-CTR groups.

The extraction of DNA of all decellularized groups is summarized in Figure 6. The quantification of extracted DNA showed a significant loss of DNA in all decellularized groups when compared with the NAT group. Comparing both decellularized methods, DNA extracted in RS-CTR group was significantly lower than in SD-CTR group (*p* < 0.05, Figure 6). In this study, we have tried to corroborate the removal of DNA also in GP crosslinked ANGs without success. Apparently, the use of GP (0.10% and 0.25%) affected the enzyme digestion process and thus the purification of DNA resulting in unreliable data (data not shown).

### 2.3. Mechanical Characterization of Nerve Substitutes

**Figure 7:**
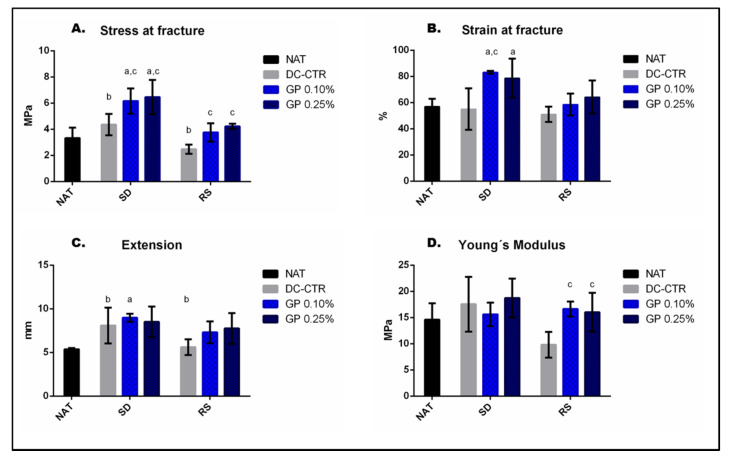
Graphic representation of tensile test results of native nerves and ANGs. Note that DC-CTR indicated the SD or RS non-crosslinked ANGs values. These analyses clearly demonstrated the impact of nerve tissue decellularization (SD and RS procedures) and subsequent GP crosslinking on the stress at fracture (**A**), strain at fracture (**B**), extension (**C**) and Young’s Modulus (**D**) values of the ANGs as well as the increase of most of these biomechanical parameters with the use of GP. Statistically significant differences (*p* < 0.05) were determined with Mann–Whitney test as follows: a = statistically significant differences vs. NAT group. b = statistically significant differences between SD-CTR vs. RS-CTR groups. c = statistically significant differences vs. its corresponding DC-CTR group. Finally, no significant differences were observed when GP 0.10% values were compared to GP 0.25% in SD and RS ANGs (*p* > 0.05).

To analyze the biomechanical properties of native, ANGs and GP-ANGs the tensile test was performed and results are summarized in Table 1 and Figure 7.

The analysis of the stress at fracture values (Figure 7A) showed no significant (*p* > 0.05) differences between SD-CTR and RS-CTR groups and native group, but differences between SD-CTR and RS-CTR groups were statistically significant (*p* < 0.05). In addition, GP crosslinked ANGs (0.10 and 0.25%) showed significantly higher stress at fracture values (*p* < 0.05) than their corresponding decellularized control group, which in the case of both SD-GP groups differences were significant as compared to NAT group (*p* < 0.05).

Besides, significantly higher values of both SD-GP groups (*p* < 0.05) were observed in strain at fracture when they were compared with the native group (Figure 7B). Extension values were significantly lower in RS-CTR than SD-CTR group and SD-GP 0.10% presented significant differences with native group (Figure 7C). Finally, both RS-GP groups were significantly higher than its decellularized control group in Young´s Modulus values (*p* < 0.05, Figure 7D).

No significant differences (*p* > 0.05) were obtained with the use of 0.10% or 0.25% GP concentrations within each decellularization technique at the biomechanical level in any test.

### 2.4. Ex-Vivo Biocompatibility

The impact of the decellularization methods and GP crosslinking on the rat adipose-derived mesenchymal stem cells (rAMSC)-biomaterial interactions, or ex vivo biocompatibility, were determined at 48 h with Live/Dead^®^ cell viability (L/D), Water soluble tetrazolium-1 (WST-1), and released DNA assays.

**Figure 8:**
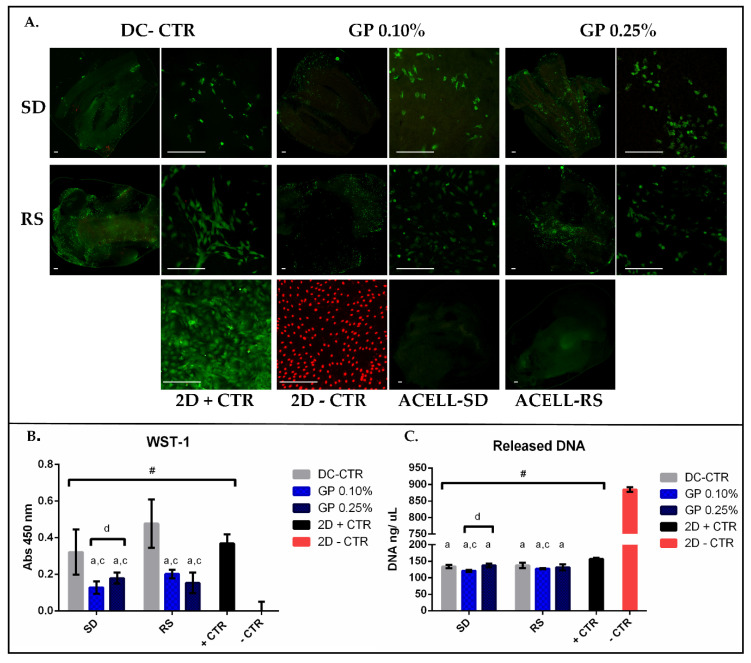
Ex vivo cell-biomaterials interaction profile. The behavior of rAMSC cultured within ANGs is shown for (**A**) Live&Dead (L/D) analysis. Scale bar = 200 µm; (**B**) WST-1; (**C**) quantification of released DNA. Decellularized nerves plotted as a function of Sondell (SD) or Roosens (RS) decellularization methods and concentration of GP used. In addition, non-crosslinked procedures were indicated as decellularized control (DC-CTR). 2D negative and positive controls were indicated as 2D -CTR and 2D +CTR respectively. For L/D (**A**) assay non-cell seeded ANGs of each decellularization methods (ACELL SD and RS respectively) were included as technical controls confirming that these analyses were conducted with cell-free biomaterials. For B and C mean values with their respective error bars correspond to standard deviations were graphed. Statistically significant differences (*p* < 0.05) were determined with Mann–Whitney test as follows: # = statistically significant differences vs. 2D—CTR. a = statistically significant differences vs. 2D + CTR. c = statistically significant differences vs. its corresponding DC-CTR group. d = statistically significant differences between GP 0.10% vs. GP 0.25% concentration used in each decellularized condition (SD or RS).

The morphofunctional L/D assay demonstrated the presence of viable cells attached to the surface of all ANGs generated (Figure 8). Moreover, dead cells (red fluorescence) were clearly observed in the 2D negative control group as expected (Figure 8). In SD groups, viable cells were found in lower quantity than RS groups. Furthermore, cells tended to be more elongated, thus comparable to 2D positive control group, in RS-CTR group than the other experimental conditions. It should be noted that the crosslinking with GP (0.10% and 0.25%), independently of the decellularization procedure used, minimally affected the attachment of the viable cells to the ANGs surface. In the case of SD group, no clear differences were observed between SD-CTR group and SD-GP groups. However, when GP concentration was increased from 0.10 to 0.25% in RS groups, cells tended to lose their fusiform morphology and shorter cytoplasmic extensions were observed (Figure 8). Besides, only a few dead cells were observed within all ANGs generated. It is probably that dead cells, unable to attach to the biomaterials, were removed during the staining process.

With WST-1 assay (data summarized in Table 2) it was possible to observe high level of cell metabolic activity within SD and RS-CTR groups, these values being comparable to the technical 2D positive control group (Figure 8). Additionally, WST-1 revealed a significant reduction (*p* < 0.05) of the absorbance with the use of GP as compared to their controls (SD-CTR or RS-CTR respectively) (Figure 8). When WST-1 values were compared between both GP concentrations used within the ANGs, differences were only significant between SD-GP 0.10% vs. SD-GP 0.25% (*p* = 0.12) groups.

The quantification of the DNA (data summarized in Table 2) demonstrated significantly lower values in experimental conditions as compared to the 2D negative control group (*p* < 0.05), where the 100% of the DNA was released (Figure 8C). In addition, DNA values were significantly higher in 2D positive control group as compared to all ANGs generated (Figure 8C). When DNA values were analyzed in SD groups, only SD-GP 0.10% group showed significantly lower values than SD-CTR and SD-GP 0.25% groups respectively (*p* < 0.05). Similar results were observed in RS groups, where differences were only significant between RS-CTR and RS-GP 0.10% (*p* < 0.05) (Figure 8C).

## 3. Discussion

The treatment of peripheral nerve injuries remains as a partially resolved issue in reconstructive surgery and engineered substitutes, especially ANGs, appeared as one of the most promising alternatives in this field [7,22]. In this context, the aim of this study was to generate novel biocompatible ANGs with enhanced biomechanical and structural properties. Here, SD and RS decellularization methods were conducted to generate ANGs which were subsequently treated with GP as a chemical crosslinker agent. This work is one of the first in which GP was used as a chemical agent to improve the biomechanical properties of ANGs and therefore a comprehensive ex vivo characterization of their histological, ultrastructural, biomechanical, and biological properties was carried out.

The success of decellularization techniques, as compared to conventional tissue engineering biofabrication processes, lies in the possibility to generate non-immunogenic, biocompatible, and proregenerative tissue-specific acellular matrices [41]. However, it is well-documented that decellularization methods have an irreversible impact on the molecular composition and even on the structure of the remaining ECM [7,20,21,42]. In the case of ANGs, SD method is one of the most widely used for this purpose [7]. This detergent-based technique combines the use of 4% sodium deoxycholate and 3% Triton X-100 resulting in a relatively efficient cell lysis and ECM preservation [40] with demonstrated in vivo biocompatibility and nerve tissue regeneration [7,22]. More recently, our research group introduced the RS method, originally used in cardiac tissue engineering [20], to generate ANGs. This technique combines 1% Triton X-100 with enzymatic digestions (DNase, RNase and trypsin) resulting in a more efficient decellularization and ECM preservation than conventional SD procedure [21].

In this opportunity, the DNA evaluation, histology, and SEM analyses confirmed the differences between SD and RS methods. Indeed, RS technique resulted in a lower DNA content, better decellularization and ECM structural preservation than SD-based ANGs, being these new findings in concordance with the previous studies [21,22]. In the present work, the subsequent chemical treatment of SD and RS ANGs with GP did not evoke remarkable histological or ultrastructural changes obtaining in both cases a well-preserved 3D histological pattern. Furthermore, DAPI and immunohistochemical analyses (S-100, VIM and NFL) accurately confirmed the efficient removal of DNA and cellular remnants. Curiously, histology revealed a bluish reaction (due to the use of GP) in the endoneurial compartment of RS-GP groups, especially in RS-GP0.25%, but this reaction did not occur in any SD-GP group. These findings are probably related to a better preservation of the ECM in RS groups and particularly to the presence of myelin debris which were confirmed by MCOLL histochemical method and SEM analyses. These myelin debris probably will induce the recruitment of macrophages to remove these debris initiating a complex process (which could take weeks) which could delay the nerve tissue regeneration [2,43]. Probably, the presence of myelin debris could be an explanation why the use of RS ANGs in nerve repair were comparable, but not superior to the autograft technique [22]. Further studies will determine the implications of the presence of myelin debris within the novel GP-crosslinked RS ANGs in nerve repair.

Concerning the histochemical and quantitative analyses of the ECM, AB staining demonstrated a significant, but beneficial reduction of proteoglycans with SD and RS methods. Although it confirms an alteration of the remaining ECM, in the case of ANGs it is important to remove these non-fibrillar molecules, generally through the use of chondroitinase ABC [7]. It is well-known that chondroitin sulfate-rich proteoglycans can hinder growth-promoting ECM cues such as laminin resulting in a decreased, delayed, or even inhibition of nerve tissue regeneration [7,19,44]. Moreover, the use of GP reduced the proteoglycans histochemical reaction. Probably, GP was able to chemically interact with the remaining proteoglycans reducing the binding sites for the AB cationic dye. The histochemical and quantitative analysis of the collagen network, conducted with PS histochemical staining, also demonstrated a significant reduction and 3D organization of these ECM fibers in ANGs as compared to native condition. The reduction was more evident with the use of RS procedure and the crosslinking with GP did not result in noteworthy histochemical and ultrastructural changes. As mentioned above, laminin plays key roles during peripheral nerve regeneration, this basal membrane glycoprotein supports the Schwann cells migration, proliferation, and axonal growth process [45,46]. Therefore, methods of decellularization that adequately preserve these glycoproteins are recommended [19]. In this study, the immunohistochemical analysis of laminin demonstrated that RS procedure resulted in a considerably better preservation and 3D organization of this glycoprotein at the endoneurial compartment than SD procedure being not affected by the subsequent treatment with GP. From the structural and/or molecular point of view, our novel GP crosslinked ANGs, especially those generated with RS method could provide physical and molecular cues for nerve regenerative microenvironment.

From the structural and biomechanical point of view, it is well-known that substitutes generated by tissue engineering to repair injured nerves must be resistant, flexible, and elastic enough to be easily handled during surgical implantation [22,47]. However, meeting these basic requirements is still a challenge, actually most of the decellularization procedures used to generate ANGs have a negative impact on the biomechanical properties making them more difficult to handle and implant during nerve reconstruction [7,18,19,21,44]. In this study, the tensile test confirms that native nerves are biomechanically delicate organs and its biomechanical properties were moderately affected by both decellularization procedures, especially those generated with SD method, which were more rigid as previously demonstrated [21]. In order to compensate the biomechanical detriment caused by decellularization process, SD and RS ANGs were crosslinked with GP. This is a natural iridoid glycosides isolated from the fruit of *Gardenia Jasminoides* [48] and often used to enhance the biomechanical properties of biomaterials in tissue engineering [32] including guidance conduits for nerve repair [38]. In this regard, the use of GP within ANGs resulted in a significant improvement of the biomechanical resistance at fracture keeping an adequate range of viscoelastic (Young’s Modulus) and deformation (strain at fracture and extension) properties. These results were considerable more favorable and comparable to native nerves with GP-crosslinked RS ANGs, where no significant differences were obtained between both concentrations of GP used. Furthermore, our results suggest a relation between the degree of ECM preservation and the biomechanical properties of the ANGs generated. In fact, in the case of SD, the increase of the rigidity could be attributed to the use of sodium deoxycholate which considerably decreased the proteoglycans content and thus the hydration rate, but preserved an abundant collagen network. In this context, GP could spontaneously react with primary amino groups of the remaining collagen fibers via imide crosslinking mechanism as previously suggested [49,50]. Moreover, the tensile characterization of our ANGs registered considerably better biomechanical values than other previously described engineered nerve substitutes [51]. From the biomechanical standpoint, our results support the idea that our novel GP crosslinked RS ANGs have a range of biomechanical properties very similar to the native nerves, which make them suitable for potential use in nerve repair.

Currently, technical progress in tissue engineering allows to design and generate highly complex 3D substitutes for tissue repair and replacement [52,53]. However, most of them are based on the use of synthetic biomaterials which are less biocompatible and lack pro-regenerative properties [54,55]. In this context, ANGs are composed by a natural ECM with well-known pro regenerative molecules, such as collagen and laminin which were not structurally affected by the treatment with GP. In general, the use of cytotoxic agents during the decellularization process could have an impact on the biocompatibility of the ANGs [18,19]. In this comparative study, the cell-biomaterials interaction analyses confirmed that a high number of viable and metabolically active rADMSCs were able to adhere, grow, and acquire an elongated morphology within our ANGs. Even better outcomes were achieved when ANGs were generated with RS method, being these results in concordance with previous histological and hematological findings of a recent in vivo study [22]. The treatment with GP, slightly decreased the cell viability and functionality of the rADMSCs cultured within these matrices, particularly when using 0.25% of GP. Despite the impact of GP on the cell functionality, overall results were considerably acceptable and better when RS ANGs were crosslinked with 0.10% of GP. These analyses point out that the decellularization method is a determinant factor in the biocompatibility properties of the ANGs generated, which can be further affected by the crosslinking agent used. The reduction of the biological properties of the crosslinked ANGs may be related to GP-induced molecular changes on the remaining ECM (reduction of the cell-ECM interaction sites) or to certain degree of cytotoxicity. In this regard, further studies could be needed to determine whether this is due to a cytotoxic effect of GP or whether the cells need a longer adaptation period to be able to grow within the new 3D microenvironment as observed after the modification of the structural properties of other biomaterials [51]. Nevertheless, the potential use of these novel GP crosslinked ANGs in nerve repair is supported by previous studies where GP crosslinked biomaterials were successfully used in vivo with acceptable degree of biocompatibility and tissue regeneration [30,37,53,56,57].

Based on the overall results obtained in this study we hypothesize that our ANGs crosslinked with GP could represent a promising alternative in nerve repair. These novel substitutes, as well as the conventional ANGs, could be generated from a wide range of donor nerves. In addition, GP could improve the biostability of these ANGs generated, as was previously demonstrated in other decellularized tissues [31,58,59], and keep stored under sterile condition until use. Therefore, these products could help solve some of the problems associated to the nerve autograft technique, which is not always available and donor sensory nerves rarely respond to the anatomical needs [47]. As compared to the nerve allografts which has a high risk of rejection or immunological response, our ANGs are considerably less immunogenic because of their efficient degree of decellularization. Furthermore, the subsequent treatment with GP, could provide anti-inflammatory properties to our grafts as previously demonstrated by other authors [22,37,38]. In addition, crosslinked ANGs have improved biomechanical properties as compared to the conventional ANGs, which are easier to handle and suture. Once implanted, their improved resistance and adequate flexibility could help reduce the biomechanical damage, often caused by rigid biomedical devices [1,60], to the regenerating and surrounding tissues. Moreover, their viscoelastic properties could decrease the risk of post-surgical tension, rupture, or compression which have been observed with the use of nerve conduits [61,62]. These novel ANGs, especially those generated with RS protocol and treated with GP are composed by a natural tissue-specific ECM with a well-preserved collagen network and laminin-based endoneurial tubes supporting their future use in nerve repair and regeneration. Moreover, the improvements obtained with GP could contribute by enhancing the physical, structural, and/or biological stability of the ANGs once stored. Nonetheless, it is still necessary to determine the optimal long-term store conditions of these advanced therapy products.

## 4. Materials and Methods

### 4.1. Nerve Isolation and Decellularization Process

PNs samples were obtained from 35 adult Wistar rats of 12 weeks of age. The animals were kept during the whole study in the Experimental Unit of the University Hospital Virgen de las Nieves in Granada (Spain). To obtain the sciatic nerves, the animals were deeply anaesthetized [intraperitoneal injection of acepromazine (Calmo-Neosan^®^ 0.001 mg/g), ketamine (Imalgene 1000^®^ 0.15 mg/g) and atropine (0.05 µg/g)] and euthanized with Eutanex^®^ solution. Subsequently, ~3 cm of both sciatic nerves (*n* = 70 nerves of 3 cm each) were removed and cryopreserved (10% DMSO in fetal bovine serum at −80 °C) until use. A total of 60 PNs were defrosted at room temperature (RT), washed with 0.1 M phosphate buffered saline (PBS) (pH = 7), sectioned into 1 cm segments and randomly assigned to the SD or RS decellularization techniques generating SD and RS-ANGs respectively. Exceptions were made for biomechanical tests (see Section 4.7).

Briefly, to generate the SD-ANGs nerve segments were treated as follow: (i) distilled water (7 h); (ii) 3% Triton X-100 (T8787, Sigma-Aldrich, Steinheim, Germany) over night (ON, 16–18 h); (iii) 4% sodium deoxycholate (D6750, Sigma-Aldrich, Steinheim, Germany ) for 24 h. The complete procedure was repeated and then nerves were abundantly washed in distilled water and kept in PBS at 4 °C until further use. The whole procedure was conducted at room temperature and using constant agitation (24 rpm) [40].

For the generation of RS-ANGs the following procedures were carried out: (i) immersion in 50 mM Tris buffer (pH = 8) at 4 °C ON with constant agitation (the same as SD); (ii) treatment with 1% Triton X-100 (diluted in the same buffer) at 4 °C during 24 h; (iii) profuse rinse in Hank’s balanced salt solution (HBSS); (iv) 2× consecutive treatments with an enzymatic digestion solution [100 mg/L DNase (DN25, Sigma-Aldrich, Steinheim, Germany), 20 mg/L RNase (R4875, Sigma-Aldrich, Steinheim, Germany) and 100 mg/L trypsin (T7309, Sigma-Aldrich, Steinheim, Germany) in HBSS] at 37 °C for 45 min and under constant agitation; (v) 1% Triton X-100 in 50 mM Tris buffer at 4 °C ON with constant agitation; (vi) several washes in HBSS. ANGs were kept in HBSS at 4 °C until use [20,21].

Animal procedures were conducted according to the Spanish and European regulations for animal experimentation (EU directive No. 63/2010, RD 53/2013) and approved by Ethics and Animal Experimentation Committee of Granada University (Nº 03-7-15-311 and approved at 2 September 2015), Grant Nº FIS PI14-1343.

### 4.2. Genipin Crosslinking and Experimental Groups

A total of 120 ANGs were subjected to chemical crosslinking with GP at 0.10 and 0.25% (*w*/*v*) diluted in PBS (0.1 M, pH 7.2–7.4) as described previously [32]. Briefly, ANGs were immersed in a minimum of 50 mL of GP solutions per 10 pieces of nerve of 1 cm for 72 h at 37 °C in protecting them from light. After this period, crosslinked ANGs were washed several times during 48 h with PBS and kept in this solution at 4 °C until further analyses. Finally, the experimental groups were defined based on the decellularization method (SD or RS) and the concentration of GP solution used (GP 0.10% or GP 0.25%). Moreover, SD and RS ANGs as well as native sciatic nerve were used as controls. Groups were abbreviated as follows: SD control group (SD-CTR); SD crosslinked with 0.10% GP solution (SD-GP 0.10%); SD crosslinked with 0.25% GP solution (SD-GP 0.25%); RS control group (RS-CTR); RS crosslinked with 0.10% GP solution (RS-GP 0.10%); RS crosslinked with 0.25% GP solution (RS-GP 0.25%); native control group (NAT) (*n* = 30 each).

### 4.3. Histological Analyses

Peripheral nerves samples from all experimental groups and controls (*n* = 3) were fixed for 48 h in 10% neutral buffered formalin solution at RT, washed, dehydrated, cleared and finally transversally embedded in paraffin [63]. Embedded tissues were cut at 5 μm thickness, hydrated, and subjected to a complete histological, histochemical, and immunohistochemical characterization as previously recommended [19].

To evaluate the general morphology and identify the cell nuclei, sections were stained with hematoxylin and eosin (HE). In addition, the intercalant fluorochrome 4´,6-diamidino-2-phenylindole (DAPI) was used for the identification of DNA (A-T interactions) remnants by fluorescent microscopy. In order to determine the presence and distribution of myelin sheath remnants and fibrillar collagen network sections were stained with MCOLL histochemical method as previously described [61,64]. The ECM was assessed with Alcian blue (AB) and Picrosirius (PS) histochemical methods to determine acid glycosaminoglycan and fibrillar collagen fibers respectively. In addition, the presence of the basal membrane glycoprotein laminin was determined by indirect immunohistochemistry.

In order to confirm the removal of cells and its main components, the distribution of the cytoskeletal proteins neurofilament (NFL, neuronal axons) and vimentin (VIM, fibroblast and Schwann cells), as well as S-100 protein (Schwann cells) were investigated by immunohistochemistry. The antibody used and technical details of immunohistochemical procedure are summarized in the Table 3.

### 4.4. Scanning Electron Microscopy (SEM)

PNs samples from all experimental groups and control were fixed in 2.5% of glutaraldehyde in 0.05 M cacodylate buffer (pH 7.2) at 4 °C overnight, and then washed, at least three times, in the same buffer at 4 °C. Thereafter, to preserve the myelin, the samples were post-fixed for 1 h with 2% OsO_4_. Fixed samples were dehydrated in increasing concentrations of acetone (30%, 50%, 70%, 90%, and 100%), and completely dried by using the critical point drying method [32]. Dried samples were covered with gold and analyzed under a scanning electron microscope FEI Quanta 200 using the high vacuum mode (FEI Europe, Eindhoven, The Netherlands) from the Department of Histology.

### 4.5. Quantitative Histochemical Analysis of the ECM

For these analyses, histological sections from each experimental condition stained with AB and PS staining, but without contrasts were used (*n* = 3 for each group). Stainings were photographed under the same light, aperture, and exposure parameters with a Nikon Eclipse Ti 90 microscope equipped with a Nikon DS-Ri2 digital camera (Nikon, Tokyo, Japan).

In this study, the endoneurial compartment, essential to promote nerve regeneration, was subjected to quantitative histological analyses to determine the percentage of area (area fraction) occupied by the remaining collagen or proteoglycans ECM (PS and AB staining respectively) [22,65]. Briefly, histological images were processed with the Image J software (National Institutes of Health, Bethesda, MD, USA) to select the PS and AB positive histochemical reactions (color split channel function). Afterwards, images were converted into binary format and then the area fraction was automatically calculated (in 20 areas of 7785.44 µ^2^/image) by using the measure function of the software. Area fraction values provide quantitative information concerning the density of the ECM components within the ANGs.

### 4.6. Quantification of DNA Remnants

To ensure that the samples are correctly decellularized and meet the requirements of <50 ng DNA/mg of dry weight tissue as described in the literature [19,66], total DNA was extracted from nerves samples corresponding to each experimental groups (*n* = 5) using the QIAamp DNA Mini Kit (Quiagen, Hilden, Germany) following the manufacture recommendations. Furthermore, the DNA was quantified by using a NanoDrop 2000 spectrophotometer (Thermo Fisher Scientific, Waltham, MA, USA). For these analyses five technical reads were run per sample. Finally, total DNA results were normalized to tissue dry weight of each sample.

### 4.7. Tensile Tests

In order to determine the impact of the decellularization technique and GP crosslinking, PNs were subjected to tensile test to calculate the stress, strain, and extension at fracture and Young Modulus as previously described [19,21,51]. Tensile tests were conducted with an electromechanical material testing machine (Instron, Model 5943, Needham, MA, USA) with the software Bluehill 3.62 and a 50 N charge cell load. For these tests, 4 samples of each condition (segments of ~3 cm length) were placed between the instrument grips leaving a constant distance of 1 cm [51]. These tests were run at a constant strain rate of 10 mm/min and a pre-charge value of 5·10^−3^ N at RT. Data were plotted as stress–strain curves, and stress at fracture, strain and extension at break, and Young’s modulus (initial linear portion) was calculated.

### 4.8. Ex-Vivo Biocompatibility (Cell-Biomaterial Interaction)

To study the biocompatibility of ANGs generated, the interaction of rat adipose-derived mesenchymal stem cells (rAMSC) with these acellular matrices was evaluated. First, to ensure the attachment of cells to the ANGs generated instead to the Costar^®^ 24-well cell culture plate surface (Corning, New York, NY, USA), each well was pre-coated with 300 µL of sterilized 3.5% (*w*/*v*) type I agarose (A4718, Sigma-Aldrich, Steinheim, Germany) in PBS. Second, ANGs segments of 5 mm length were longitudinally opened, placed in agarose-coated wells, and then 2 × 10^4^ rAMSCs (passage 4–6) were seeded in contact to the inner surface of each sample (endoneurial compartment). Afterwards, wells were supplemented with basal culture medium [Dulbecco’s modified Eagle medium (DMEM) supplemented with 10% fetal bovine serum (FBS) and 1% antibiotic and antimycotics commercial solution (all products from Sigma Aldrich, Steinheim, Germany)] and kept for 48 h under standard culture conditions (37 °C and 5% CO_2_). Finally, the cell-biomaterial interactions were determined by using WST-1 assay (Roche, Mannheim, Germany), Live/Dead^®^ Cell Viability Assay (L/D) (Thermo-Fisher Scientific, Portland, OR, USA), and quantification of released DNA as previously described [21,27,67] and recommended [19].

The cellular metabolic activity of the cells seeded within the different ANGs generated was quantitatively measured with the water-soluble tetrazolium salt-1 (WST-1) assay following the manufacturer and previous recommendations [27,32]. This method measures the colorimetric reaction, formazan dye formation, resulted from the mitochondrial dehydrogenase cleavage of the tetrazolium salt. Briefly, ANGs and GP crosslinked-ANGs were incubated with the working solution reagent for 4 h at 37 °C. After this period, the solution was collected and the colorimetric reaction was analyzed, in its maximum peak at 450 nm, with a spectrophotometer (ASYS UVM340) and DigiRead software (Biocrom Ltd., Cambridge, UK).

The L/D assay was performed according to the manufacturer’s recommendations. Briefly, cell seeded samples and controls were incubated with the working solution composed of calcein and ethidium homodimer-1 for 15 min at 37 °C. The nerves samples were then rinsed in PBS and analyzed under a Nikon Eclipse Ti fluorescence equipped with a Nikon DXM 1200c Digital Camera (Nikon, Tokyo, Japan). Viable and metabolically active cells incorporated the supravital fluorochrome calcein and appeared green, while dead cells, with irreversible cell membrane damage, incorporated the ethidium fluorochrome emitting red nuclear fluorescence.

The quantification of released-DNA to the supernatant culture medium was used as a marker of irreversible cellular membrane damage, as previously described [27,32,68]. Culture medium was collected in Eppendorf tubes and DNA was measured using NanoDrop 2000, performing analyses with five technical reads per sample.

In all assays, ANGs placed in pre-coated wells and supplemented with basal culture medium but without cell seeded on top were used as a negative control. Furthermore, 2 × 10^4^ rAMSCs were seeded in wells without agarose-coating and used as 2D positive or negative technical controls. In the case of the 2D negative control, an irreversible cell-membrane and nuclei damage was induced by using 2% Triton X-100. Finally, all these assays were performed in quintuplicate.

### 4.9. Statistical Analysis

In this study, all quantitative data were collected and analyzed with SPSS version 24.00 software (SPSS Inc., Chicago, IL, USA). First, all variables were subjected to the Shapiro–Wilk test of normality. As variables showed a non-normal distribution, Kruskal–Wallis non-parametric test followed by a post-hoc group-by-group analysis conducted with Mann–Whitney non-parametric test was used. In this sense, *p* < 0.05 was considered statistically significant in the two-tailed tests.

## 5. Conclusions

This ex vivo study demonstrates a superior histological pattern, ECM preservation, biomechanical properties, and biocompatibility in RS ANGs than those generated with SD procedure. The use of GP, as crosslinker agent, did not remarkably affect the typical nerve histological microarchitecture of the SD or RS ANGs generated. In fact, our results indicate that GP contributed to a better preservation of the ECM of SD or RS ANGs generated. The biomechanical analyses demonstrated that GP turned out to be an efficient crosslinking agent to improve the stiffness and viscoelastic properties of our ANGs. Even better outcomes were achieved when GP was used in combination with RS ANGs. In addition, our cell-biomaterials interaction analyses confirmed high levels of ex vivo biocompatibility in both crosslinked ANGs, especially when RS ANGs were treated with 0.10% GP. All these results suggest that these novel crosslinked ANGs could be potentially used in peripheral nerve repair. However, further in vivo preclinical studies are still required to demonstrate the therapeutic usefulness of our GP crosslinked ANGs in peripheral nerve repair.

## Figures and Tables

**Table 1:** Biomechanical analysis of native, decellularized and crosslinked ANGs. Results corresponding to each biomechanical parameter are shown as mean ± standard deviation values.

Groups	Stress at Fracture		Strain at Fracture		Extension		Young´s Modulus	
	(MPa)		(%)		(mm)		(MPa)	
NAT	3.33	±0.79	56.97	±4.68	5.99	±0.36	13.40	±3.21
SD-CTR	4.36	±0.82	55.08	±15.89	8.17	±2.06	16.70	±5.23
SD-GP 0.10%	6.16	±0.96	83.16	±1.13	8.98	±0.46	15.61	±2.25
SD-GP 0.25%	6.46	±1.31	78.64	±14.86	8.51	±1.76	18.73	±3.70
RS-CTR	2.47	±0.35	51.02	±5.82	5.61	±0.90	9.81	±2.44
RS-GP 0.10%	3.76	±0.70	58.55	±8.35	7.31	±1.24	16.63	±1.44
RS-GP 0.25%	4.22	±0.20	64.24	±12.61	7.75	±1.76	16.02	±3.69

**Table 2:** Ex vivo results of the quantitative biocompatibility analyses WST-1 and released DNA. Results obtained with these biochemical analyses are shown as mean ± standard deviation values. In addition, WST-1 and released-DNA results were normalized with their positive or negative 2D controls respectively which represented 100%.

	WST-1			Released DNA		
	(Abs 450 nm)		(%)	(ng/uL)		(%)
	Mean	±SD	Normalized	Mean	±SD	Normalized
SD-CTR	0.32	±0.12	87.35	133.94	±5.09	15.66
SD-GP 0.1%	0.13	±0.03	34.70	120.91	±2.87	14.14
SD-GP 0.25%	0.18	±0.03	48.62	137.28	±5.83	16.06
RS-CTR	0.48	±0.13	129.43	137.16	±7.88	16.04
RS-GP 0.10%	0.20	±0.02	54.81	127.41	±1.92	14.90
RS-GP 0.25%	0.15	±0.06	41.36	131.61	±9.12	15.39
2D + CTR	0.37	±0.07	100.00	127.14	±2.26	18.33
2D − CTR	0.00	±0.26	0.00	885.03	±7.11	100.00

**Table 3:** Antibodies and reagents used for immunohistochemical analyses.

Antibody/Reagent	Dilution/Incubation	Pretreatment	Reference
Rabbit Policlonal anti- S100 antibody (Z0311)	1: 400 Overnight at 4 °C	Citrate buffer pH = 630 min at 95 °C	DakoCytomation, Glostrup, Denmark (ref. Z0311)
Mouse Monoclonal Neurofilament	1: 500 1 h at RT	EDTA buffer pH = 8 25 min at 95 °C	Sigma-Aldrich N2912, Steinheim, Germany (ref. RMdO20)
Mouse anti-vimentin monoclonal clone V9	1:200 1 h at RT	Citrate buffer pH = 6 25 min at 95 °C	Sigma-Aldrich. St. Louis, MO, USA (ref. V6630)
Rabbit anti-laminin polyclonal	1:200 Overnight at 4 °C	Citrate buffer pH = 6 25 min at 95 °C	Abcam. Cambridge, UK (ref. ab11575)
ImmPRESS^®^ HRP Anti-Mouse IgG (Peroxidase)	Ready to use 30 min at RT	-	Vector Laboratories. Burlingame, EEUU (ref. MP-7402)
ImmPRESS^®^ HRP Anti-Rabbit IgG (Peroxidase)	Ready to use 30 min at RT	-	Vector Laboratories. Burlingame, EEUU (ref. MP-7401)
Chromogen: Diaminobenzidine ready to use kit	-	-	Vector Laboratories. Burlingame, EEUU (ref. SK-4100)
Contrast: Harris Hematoxylin	30 s	-	Thermo Scientific. Runcorn, UK (ref. 6765004)

## Abbreviations

PNs	Peripheral nerves
ECM	Extra cellular matrix
ANGs	Acellular nerve grafts
GP	Genipin
SD	Sondell
RS	Roosens
HD	Hudson-based
RT	Room temperature
PBS	Phosphate buffer saline
HBSS	Hank´s balance salt solution
SD-ANGs	Acellular nerve allografts decellularized by Sondell method
RS-ANGs	Acellular nerve allografts decellularized by Roosens method
SD-CTR	Sondell non-crosslinked decellularized control group
SD-GP 0.10%	Sondell 0.10% genipin crosslinked decellularized group
SD-GP 0.25%	Sondell 0.25% genipin crosslinked decellularized group
RS-CTR	Roosens non-crosslinked decellularized control group
RS-GP 0.10%	Roosens 0.10% genipin crosslinked decellularized group
RS-GP 0.25%	Roosens 0.25% genipin crosslinked decellularized group
NAT	Native nerve control group
HE	Hematoxylin & eosin
DAPI	4´,6-diamidino-2-phenylindole
AB	Alcian blue
PS	Picrosirius
SEM	Surface electron microscopy
rAMSC	Rat adipose-derived mesenchymal stem cells
DMEM	Dulbecco’s modified Eagle medium
FBS	Fetal bovine serum
WST-1	Water soluble tetrazolium-1
L/D	Live/Dead^®^ cell viability assay
NFL	Neurofilament
VIM	Vimentin
LAM	Laminin
DC-CTR	non-crosslinked decellularized control group
GP-ANGs	Genipin crosslinked acellular nerve grafts
2D + CTR	2 dimensional positive control
2D − CTR	2 dimensional negative control
ACELL-SD	Technical control of non-cellular seeding on Sondell acellular scaffold.
ACELL-RS	Technical control of non-cellular seeding on Roosens acellular scaffold.
MCOLL	Histochemical method for simultaneous staining of myelin and collagen fibers
